# SARS-CoV-2 Contamination on Healthy Individuals' Hands in Community Settings During the COVID-19 Pandemic

**DOI:** 10.7759/cureus.54919

**Published:** 2024-02-26

**Authors:** Hidehito Matsui, Miho Sugamata, Harumi Endo, Yumiko Suzuki, Yukiko Takarabe, Yukie Yamaguchi, Rei Hokari, Aki Ishiyama, Chihiro Ueda, Eri Nakajima, Osamu Takeuchi, Atsushi Ujihara, Yasuo Imoto, Hideaki Hanaki

**Affiliations:** 1 Research Center for Infection Control, Ōmura Satoshi Memorial Institute, Kitasato University, Tokyo, JPN; 2 Research Center for Tropical Diseases, Ōmura Satoshi Memorial Institute, Kitasato University, Tokyo, JPN; 3 Research and Development, Kobe Testing Center, Japan Textile Products Quality and Technology Center, Hyogo, JPN; 4 Department of Research, Kitasato Institute Hospital, Kitasato University, Tokyo, JPN; 5 R&D Team, Kobe Testing Center, Japan Textile Products Quality and Technology Center, Hyogo, JPN

**Keywords:** healthy participants, contact transmission, hands, sars-cov-2, covid-19

## Abstract

Introduction

Hand hygiene is an infection control measure for COVID-19 in our daily lives; however, the contamination levels of SARS-CoV-2 in the hands of healthy individuals remain unclear. Thus, we aimed to evaluate SARS-CoV-2 contamination levels by detecting viral RNA and viable viruses in samples obtained from the hands of 925 healthy individuals.

Methods

Swab samples were collected from the palms and fingers of healthy participants, including office workers, public officers, university students, university faculty and staff, and hospital staff between December 2022 and March 2023. The collected swab samples were analyzed using reverse transcription-quantitative polymerase chain reaction (RT-qPCR) for SARS-CoV-2 RNA detection. Viral RNA-positive samples were subjected to plaque assay to detect viable viruses.

Results

We collected 1,022 swab samples from the hands of healthy participants. According to the criteria for data collection, 97 samples were excluded, and 925 samples were analyzed using RT-qPCR. SARS-CoV-2 RNA was detected in three of the 925 samples. The viral RNA detection rate was 0.32% (3/925), and the viral RNA copy numbers ranged from 5.0×10^3^ to 1.7×10^5^ copies/mL. The RT-qPCR-positive samples did not contain viable viruses, as confirmed by the plaque assay results.

Conclusions

The detection rate of SARS-CoV-2 RNA from the hands of healthy individuals was extremely low, and no viable viruses were detected. These results suggest that the risk of contact transmission via hands in a community setting is extremely rare.

## Introduction

The COVID-19 pandemic led to significant changes in infection control measures for the general public in their daily lives. The potential transmission routes of SARS-CoV-2 are droplets and aerosols containing the virus and contact transmission through fomites [1,2]. The infection control measures in their daily lives against COVID-19 include wearing masks, indoor air ventilation, and hand hygiene. Wearing masks decreases the spread and uptake of droplets and aerosols containing the virus in a simulated airborne transmission system [3]. A systematic review of mask usage in community settings has also reported that it contributes to a decrease in the incidence, hospitalization, and mortality of COVID-19 [4]. Indoor air ventilation has been recommended to reduce the transmission of SARS-CoV-2, and the Centers for Disease Control and Prevention in the United States announced ventilation guidelines in May 2023 [5]. Hand hygiene practices to prevent COVID-19 transmission have been adopted in several situations, such as when returning home from public places and when touching objects that are considered fomites in daily life. Alcohols and surfactants have been used as effective disinfectants for hand hygiene; they act through the disruption of the viral envelope. Ethanol and 2-propanol have demonstrated high efficacy in deactivating SARS-CoV-2 at concentrations ≥30% and reducing the infectious titer of SARS-CoV-2 by over 5.9 log10 units within 0.5 min [6]. Commercially available alcohol-based sanitizers can effectively inactivate SARS-CoV-2 [7,8]. Although the frequency of hand disinfection or washing has excessively increased, the extent to which the hands of healthy individuals are contaminated with SARS-CoV-2 in their daily lives during the COVID-19 pandemic is unclear. Therefore, we aimed to investigate the actual contamination levels of SARS-CoV-2 on hand surfaces of healthy individuals by detecting viral RNA and viable viruses.

## Materials and methods

Sample collection from palms and fingers

Samples were collected from the palms and fingers of the study participants’ two hands between December 2022 and March 2023. Healthy individuals aged ≥18 years with no fever or other symptoms of infectious diseases were included in the study. Participants were recruited from the university's relevant parties, healthcare and non-healthcare staff of the hospital, office workers, and public officers. After obtaining consent from the participants, we collected information about their age, sex, occupation, and COVID-19 infection history, as well as the COVID-19 infection history of their housemates within one month before sample collection. Samples were collected from the inner surfaces of both hands by rubbing the inner surfaces of all fingers two times and continuously rubbing the whole palms four to five times, each using two sterile polyester swabs simultaneously (Japan Cotton Buds Industry Limited, Tokyo, Japan). One swab was suspended in 0.5 mL of sterile saline for reverse transcription-quantitative polymerase chain reaction (RT-qPCR) to detect viral RNA. The other swab was suspended in 1.5 mL of universal transport medium (UTM) (Copan Diagnostics, Murrieta, USA) for the plaque assay. Swab samples were then stored at −80°C until further use. This study was reviewed and approved by the Research Ethics Committee of the Kitasato Institute Hospital (Approval no. 22040).

Detection of SARS-CoV-2 RNA by RT-qPCR

The swab samples suspended in saline were directly subjected to RT-qPCR for the detection of SARS-CoV-2 RNA using the SARS-CoV-2 N2 gene detection kit (Toyobo Co., Ltd., Osaka, Japan), according to the manufacturer’s protocol. A dilution series of 10^4^ to 10^6^ copies/mL of SARS-CoV-2 positive control RNA (Nihon Gene Research Laboratories, Inc., Miyagi, Japan) was prepared to obtain a standard curve, and the viral RNA copy numbers in the samples were calculated. The detection limit of RT-qPCR was 10^3^ copies/mL [9].

Plaque assay for detecting viable SARS-CoV-2

The RT-qPCR-positive samples were examined to confirm the presence of SARS-CoV-2. VeroE6/TMPRSS2 cells (JCRB1819) (JCRB Cell Bank, Osaka, Japan) [10] were cultured in a Dulbecco's modified Eagle medium (DMEM) (low glucose) (Thermo Fisher Scientific, Inc., Waltham, MA) containing 5% fetal bovine serum and 1 mg/mL geneticin (G418; Fujifilm Wako Pure Chemical Corp., Osaka, Japan) at 37°C with 5% CO_2_ until reaching subconfluency. The cells were spread at a density of 6.5 × 10^5^ cells/well in six-well plates and incubated for three days to prepare the cell monolayers. The collected swab suspensions in UTM were filtered using a 0.22-µm membrane filter, and 0.1 mL of the sample was then added to 10 wells. After incubation at 37°C with 5% CO_2_ for 1.5 hours, an overlay medium containing 2% fetal bovine serum and 0.01% diethylaminoethyl-dextran in DMEM was added. After five days of incubation, VeroE6/TMPRSS2 cells were fixed with 1% glutaraldehyde for one hour and stained with 0.0375% methylene blue to evaluate plaque formation by the virus [9].

## Results

To evaluate SARS-CoV-2 surface contamination in healthy individuals' hands, swab samples were collected between December 2022 and March 2023 in Tokyo, Japan. During this period, the seven-day moving average of new COVID-19 cases in Japan was reported to peak at approximately 180,000 cases caused by the Omicron variant at the beginning of January 2023 [11]. Thereafter, the number of new COVID-19 cases gradually declined to approximately 7,000 cases by the end of March. The rate of vaccination with second and third booster doses of the COVID-19 vaccine among Japanese people was approximately 78% and 69%, respectively, in March 2023 [12]. Throughout the study period, samples were collected from 1,022 healthy individuals. However, 97 samples were excluded because handwashing or hand antisepsis was performed within one hour before sampling. Consequently, 475 samples were collected from office workers and public officers (Table 1), and 268 and 182 samples were collected from the university and hospital personnel, respectively. Thus, the total sample size was calculated as 925. The COVID-19 infection history of participants and their housemates within one month before sample collection was examined, and participants were categorized as follows: (i) participants who were infected alone (n=13), (ii) participants whose housemates were infected alone (n=11), (iii) participants coinfected with their housemates (n=7), and (iv) participants and housemates with no reported infection (n=894).

**Table 1:** COVID-19 infection history of study participants and their housemates within one month before sampling.

Category (number of samples)		COVID-19 infection history (number of participants)				Total
		Participant alone	Housemate alone	Both participant and housemate	None	
Company and public office(n=475)						
	Office worker, Company A	1	-	1	144	146
	Office worker, Company B	1	1	1	105	108
	Office worker, Company C	-	1	-	101	102
	Public officer	1	-	-	118	119
University(n=268)						
	University faculty and staff	3	2	3	131	139
	University student	1	3	-	90	94
	Others	2	1	-	32	35
Hospital(n=182)						
	Nurse	2	-	2	44	48
	Pharmacist	-	-	-	21	21
	Clinical laboratory technologist	1	-	-	16	17
	Physical therapist or occupational therapist	-	-	-	14	14
	Doctor	-	-	-	5	5
	Clinical engineering technologist	-	-	-	1	1
	Non-healthcare workers	1	3	-	72	76
Total		13	11	7	894	925

Office workers and public officers aged 22-75 years (mean age: 44.9 years) accounted for 475 samples, with 286 (60.2%) males and 189 (39.8%) females. Testing using RT-qPCR for SARS-CoV-2 RNA identified only one positive sample collected from an office worker of Company A (Table 2). Subsequent sample collection targeted younger individuals, mainly students from a single university. Out of the 268 samples collected, 108 (40.3%) were from individuals aged ≤29 years. The age of the university participants ranged from 19 to 68 years, with a mean age of 39.7 years. Two RT-qPCR-positive results were detected among samples from the university faculty and staff (Table 3). Other samples were collected from hospital workers, including nurses, pharmacists, clinical laboratory technologists, physical therapists, occupational therapists, doctors, and clinical engineering technologists. No RT-qPCR-positive results were observed among samples from the 106 healthcare workers and the 76 non-healthcare workers in the hospital (Table 4).

**Table 2:** SARS-CoV-2 RNA detection from the hands of office workers and public officers.

Category	Sex	Age						Total number of samples	Number of SARS-CoV-2 RNA-positive samples
		20s	30s	40s	50s	60s	70s		
Office worker, Company A	Male	7	12	39	25	4	1	88	1
	Female	21	16	14	6	1	-	58	0
Office worker, Company B	Male	2	23	25	27	2	-	79	0
	Female	2	7	12	4	4	-	29	0
Office worker, Company C	Male	7	10	15	22	3	1	58	0
	Female	6	11	13	12	2	-	44	0
Public officer	Male	3	16	16	14	12	-	61	0
	Female	5	10	13	23	5	2	58	0
Total		53	105	147	133	33	4	475	1

**Table 3:** SARS-CoV-2 RNA detection from the hands of university faculty, staff, and students.

Category	Sex	Age						Total number of samples	Number of SARS-CoV-2 RNA-positive samples
		10s	20s	30s	40s	50s	60s		
University faculty and staff	Male	-	-	22	30	14	11	77	0
	Female	-	12	15	15	18	2	62	2
University student	Male	-	42	2	-	-	-	44	0
	Female	1	48	1	-	-	-	50	0
Others	Male	1	3	5	8	5	-	22	0
	Female	-	1	2	5	4	1	13	0
Total		2	106	47	58	41	14	268	2

**Table 4:** SARS-CoV-2 RNA detection from the hands of hospital staff.

Occupation	Sex	Age					Total number of samples	Number of SARS-CoV-2 RNA-positive samples
		20s	30s	40s	50s	60s		
Nurse	Male	-	-	1	-	-	1	0
	Female	7	9	18	13	-	47	0
Pharmacist	Male	1	1	4	-	-	6	0
	Female	1	4	8	1	1	15	0
Clinical laboratory technologist	Male	-	-	1	-	1	2	0
	Female	1	-	9	4	1	15	0
Physical therapist or occupational therapist	Male	2	2	2	2	-	8	0
	Female	3	2	1	-	-	6	0
Doctor	Male	-	1	1	1	-	3	0
	Female	-	1	-	-	1	2	0
Clinical engineering technologist	Male	-	-	-	-	-	0	0
	Female	1	-	-	-	-	1	0
Non-healthcare worker	Male	2	2	8	5	5	22	0
	Female	7	16	20	11	-	54	0
Total		25	38	73	37	9	182	0

The above data showed that the detection rate of SARS-CoV-2 RNA in the hands of healthy individuals was 0.32% (95% CI: 0.063-0.997). The three RT-qPCR-positive samples contained 5.0 × 10^3^ to 1.7 × 10^5^ copies/mL of SARS-CoV-2 RNA (Table 5). Although one university faculty member showed viral RNA positivity and had experienced COVID-19 infection within one month before sampling, neither the other two participants nor their housemates had a history of COVID-19 infection. The two university faculty members who showed positive results for SARS-CoV-2 RNA worked in the same department. Notably, no viable viruses were detected in RT-qPCR-positive samples, as confirmed by the plaque assay.

**Table 5:** Characteristics of SARS-CoV-2 RNA-positive samples. RT-qPCR: reverse transcription-quantitative polymerase chain reaction; Ct: cycle threshold value in RT-qPCR; PFU: plaque-forming units

Sample	Sex	Age, years	History of COVID-19 infection within one month before sampling	RT-qPCR		Viable virus (PFU/mL)
				Ct	Viral RNA (copies/mL)	
Office worker, Company A	Male	75	None	32.9	1.7×10^5^	<1
University faculty and staff	Female	60	Participant	36.4	3.0×10^4^	<1
University faculty and staff	Female	56	None	38.4	5.0×10^3^	<1

## Discussion

To reduce the risk of transmission of SARS-CoV-2 among the general public during daily life activities, several infection control measures have been implemented, such as wearing masks, ventilation of indoor air, maintaining hand hygiene, and avoiding closed spaces, crowded places, and close contact settings. To assess the efficiency of these infection control measures, it is important to identify where viable SARS-CoV-2 exists, rather than viral RNA debris. In this study, we investigated the SARS-CoV-2 contamination level in the hands of healthy people during the COVID-19 pandemic and found a low detection rate of viral RNA and no viable virus in the participants’ hands.

Hand contamination by SARS-CoV-2 in patients with COVID-19 and healthcare workers caring for patients with COVID-19 has been previously reported. In a previous study, seven out of 16 samples from the hands of the participants were found positive [13], and the detection rate was 43.8%. Although these SARS-CoV-2 RNA-positive samples were not examined for viable viruses, the cycle threshold (Ct) values of the seven samples were over 30, and four samples presented Ct values over 36. Samples collected from healthcare workers in France during the Delta variant spread in 2021 showed SARS-CoV-2 RNA positivity in two out of 192 samples (1.0%) and two positive samples from a nurse and nursing assistant caring for patients with COVID-19 presented Ct values of over 37 [14]. Similar to the results of this study, these previous studies on individuals with expected high-level exposure to SARS-CoV-2 reported low levels of hand contamination. The stability of SARS-CoV-2 on human skin was reported to be lower than that on other environmental surface materials (e.g., stainless steel, glass, and plastic) according to a study evaluating forensic autopsy human skin specimens [15,16]. The lower stability of viable SARS-CoV-2 on human skin than that on other materials may have contributed to the lower detection rate. Moreover, we reported that environmental surfaces in concert halls and banquet rooms where many people gather and homes where patients with COVID-19 stayed for recuperation had no viable virus contamination [9,17]. Moreover, the viable virus detection rate from environmental surfaces, including hospital rooms of patients with COVID-19, was 0.47% [18]. From the above data and the results of this study, fomite transmission via the human hand may entail a lower risk, compared with other COVID-19 transmission routes, in a community setting.

This study had a few limitations. First, swab samples were collected from the hands of participants belonging to only one university, one hospital, one public office, and three companies in Tokyo, Japan. Thus, it is necessary to collect samples from a more diverse population, including children and older persons living in different areas or countries with different COVID-19 infection rates and hand hygiene practices, to better understand the generalizability of our conclusions. Second, although we excluded samples that were collected from those who performed hand sanitization or handwashing one hour before sample collection, the study was conducted during the COVID-19 pandemic, a period with increased adherence to hand hygiene practices. Therefore, an increase in the detection rate of SARS-CoV-2 is expected if hand hygiene practices decrease. Finally, SARS-CoV-2 RNA was detected in samples from three individuals, two of whom had no history of COVID-19 before sampling. However, it is plausible that the participants or their housemates could have been asymptomatic at the time of data collection.

## Conclusions

We investigated the level of SARS-CoV-2 contamination in the hands of 925 healthy individuals. Although viral RNA debris was identified, its frequency and copy number were extremely low, and no viable SARS-CoV-2 was detected. Thus, our results indicate that the risk of infection through contact transmission of SARS-CoV-2 via hands is relatively low. Therefore, infection control measures for COVID-19 should focus on preventing droplet and aerosol transmission.
